# Recombinant Sendai Virus Vectors as Novel Vaccine Candidates Against Animal Viruses

**DOI:** 10.3390/v17050737

**Published:** 2025-05-21

**Authors:** Álex Gómez, Ramsés Reina

**Affiliations:** 1 Departamento de Patología Animal, Universidad de Zaragoza, 177 Calle de Miguel Sevet, 50013 Zaragoza, Spain; 2 Instituto Agroalimentario de Aragón-IA2, Universidad de Zaragoza, 177 Calle de Miguel Sevet, 50013 Zaragoza, Spain; 3 Instituto de Agrobiotecnología (CSIC-Gobierno de Navarra), 123 Avenida Pamplona, 31192 Mutilva, Spain

**Keywords:** Sendai virus vector, vaccine, immune response, animals

## Abstract

Vaccination plays a pivotal role in the control and prevention of animal infectious diseases. However, no efficient and safe universal vaccines are currently registered for major pathogens such as influenza A virus, foot-and-mouth disease virus (FMDV), simian immunodeficiency virus (SIV), and small ruminant lentiviruses (SRLV). Here, we review the development of Sendai virus (SeV) vectors as a promising vaccine platform for animal diseases. Recombinant SeV vectors (rSeVv) possess several key features that make them highly suitable for developing vaccination strategies: (1) SeV has exclusively cytoplasmic replication cycle, therefore incapable of transforming host cells by integrating into the cellular genome, (2) rSeVv can accommodate large foreign gene/s inserts (~5 kb) with strong but adjustable transgene expression, (3) can be propagated to high titers in both embryonated chicken eggs and mammalian cell lines, (4) exhibits potent infectivity across a broad range of mammalian cells from different animals species, (5) undergo transient replication in the upper and lower respiratory tracts of non-natural hosts, (6) has not been associated with disease in pigs, non-humans primates, and small ruminants, ensuring a favorable safety profile, and (7) induce a robust innate and cellular immune responses. Preclinical and clinical studies using rSeVv-based vaccines against influenza A virus, FMDV, SIV, and SRLV have yielded promising results. Therefore, this review highlights the potential of rSeVv-based vaccine platforms as a valuable strategy for combating animal viruses.

## 1. The Clinical Need for New Generation Vaccines Against Animal Diseases

Vaccination is a cornerstone of infectious disease control and prevention in animals, especially in livestock species, ensuring animal health, food security, and economic stability in the primary sector [1]. Effective immunization programs not only reduce the incidence of diseases but also minimize the need for antibiotics, addressing concerns about antimicrobial resistance [2]. While vaccines have been a cornerstone of both human and veterinary medicine, contributing significantly to disease control, many of those currently used in livestock remain based on traditional formulations, either inactivated or live attenuated pathogens, which may fail to confer strong or long-lasting protection, particularly against viral infections [3]. These limitations highlight the importance of continued innovation toward next-generation vaccines that can elicit more effective and safer immune responses in livestock.

Viral vector-based vaccines have emerged as promising tools in veterinary medicine due to their capacity to induce strong humoral and cellular immunity [4]. Several such vaccines are already available for animal diseases, including adenoviruses and herpesviruses-based vaccines, with advantages in terms of safety, stability, and ease of production [4]. Among them, the Sendai virus (SeV) has garnered attention as a potential vaccine platform for veterinary use. Preclinical studies have explored recombinant SeV vectors (rSeVv) for targeting animal pathogens that currently lack efficient and secure vaccines on a global scale, such as influenza virus [5], foot-and-mouth disease virus (FMDV) [6], simian immunodeficiency virus (SIV) [7], and small ruminant lentiviruses (SRLV) [8], obtaining promising results. Therefore, rSeVv could be used to develop novel vaccines tailored to the specific challenges of veterinary medicine.

## 2. Virology of Sendai Virus

### 2.1. Taxonomy and Phylogeny of Sendai Virus

The genus *Respirovirus* belongs to the order *Mononegavirales*, family *Paramyxoviridae,* and subfamily *Paramyxovirinae* [9]. This genus includes species such as human parainfluenza virus 1 and 3 (HPIV1 and HPIV3), bovine parainfluenza virus 3 (BPIV3), and SeV [10]. SeV (murine parainfluenza virus type 1), initially called hemagglutinating virus of Japan, was isolated in 1953 in Japan [11]. It is a murine-origin virus, recognized as a major respiratory pathogen in mice, and an endemic in rodent colonies worldwide [12,13]. While most respiroviruses are host-specific, SeV infects multiple animal species, including pigs [14,15], humans [16], non-human primates [17], and sheep [18], without producing disease.

The phylogenetic classification of SeV is primarily based on variations in the fusion (F) and hemagglutinin-neuraminidase (HN) genes, which are key markers for distinguishing it from other closely related parainfluenza viruses within the *Respirovirus* genus [10,19]. As with other RNA viruses, SeV genetic variability arises from mechanisms such as error-prone replication due to its RNA large polymerase (L) protein, which lacks proofreading capabilities [20]. As a result, accumulation of point mutations, especially in the F and HN gene sequences, can potentially affect the viral infectivity, host range, and immune evasion, thus differentiating multiple SeV strains [21]. SeV-6/94 (NCBI:txid11193), SeV-Enders (NCBI:txid11194), SeV-Fushimi (NCBI:txid11195), SeV-Hamamatsu (NCBI:txid302271), SeV-Harris (NCBI:txid11196), SeV-Nagoya (NCBI:txid317654), SeV-Ohita (NCBI:txid302272), and SeV-Z (NCBI:txid11198) are some of the principal SeV strains. Variations in the HN protein receptor-binding domain can alter the viral affinity for sialic acid receptors of different species, facilitating cross-species transmission [22,23]. Additionally, L protein-mediated mRNA editing in the phosphoprotein (P) gene enhances viral adaptability to diverse hosts and environments [24,25].

### 2.2. Genome and Virion Structure of Sendai Virus

**Figure 1:**
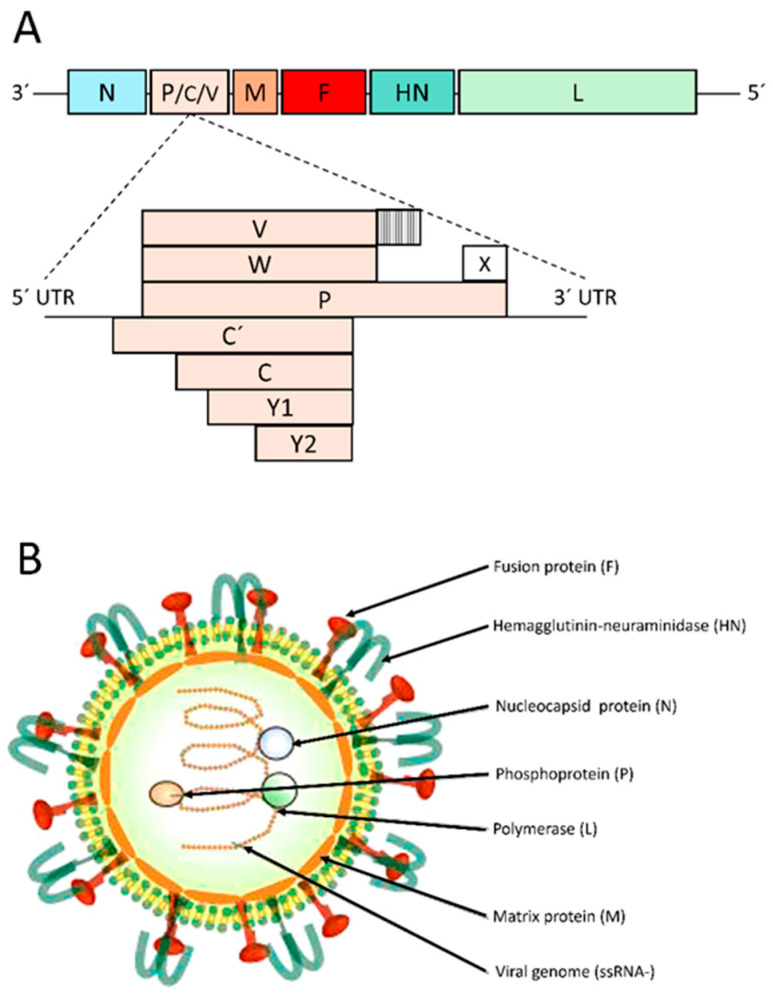
Genome and virion structure of Sendai virus (SeV). (**A**) SeV genome contains six structural genes: N-P-M-F-HN-L. The P gene encodes eight protein isoforms (P, V, W, C’, C, Y1, Y2 and X) via overlapping open reading frame and mRNA editing. (**B**) SeV virion includes the viral genome (ssRNA-), nucleocapsid protein (N), phosphoprotein (P), RNA large polymerase protein (L), matrix protein (M), hemagglutinin-neuraminidase (NH) and fusion (F) glycoproteins. Image courtesy of Dr. Lorena de Pablo-Maiso.

The SeV genome is a single-stranded, negative-sense RNA (ssRNA-) of approximately 15–16 kb in length. It consists of a 3′ leader sequence of about 50 nucleotides, followed by six structural genes arranged in the order 3′-(leader)-N-P-M-F-HN-L-(trailer)-5′ [26] (Figure 1A). The 5′ trailer region, spanning approximately 50–161 nucleotides, is essential for the transcription and replication of both the six structural genes and additional accessory proteins. Structural proteins include the surface glycoproteins HN and F, the nucleocapsid (N)-associated proteins N, P, and L, as well as the matrix protein (M) [27]. Monocistronic mRNAs of these structural genes are transcribed by the L protein. However, during transcription, the L protein occasionally undergoes polymerase slippage at a specific site within the P gene, causing the insertion of additional G residues. This process generates different mRNA variants, leading to the production of multiple protein isoforms (P, V, W, C’, C, Y1, Y2, and X) from this single gene [25,28,29]. Transcription occurs in a gradient, with the N gene being the first transcribed most frequently, followed by P, M, F, HN, and L [24]. SeV depends on the “rule of six”, which specifies that the viral genome must be hexameric in length to replicate efficiently [30].

SeV particles are pleomorphic, with an average diameter of 150–200 nm, and consist of a lipoprotein envelope surrounding a single negative unsegmented viral ssRNA- (Figure 1B).

### 2.3. Viral Cycle of Sendai Virus

**Figure 2:**
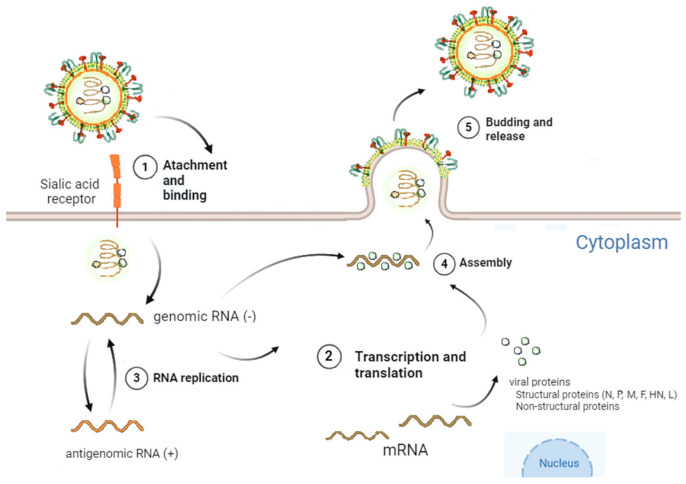
Viral cycle of the Sendai virus. (**1**) The viral cycle begins with the attachment of the virus mediated by the hemagglutinin-neuraminidase protein and is fused with the cell membrane by the fusion protein. (**2**) The nucleocapsid and genetic material are released into the cytoplasm, initiating the transcription and ending the translation into the cell ribosomes. (**3**) The viral genome is replicated by the RNA large polymerase protein to achieve the complementary antigenome. (**4**) New viral proteins and antigenomes are assembled in the Golgi apparatus. (**5**) The budding process occurs in the cell membrane where the hemagglutinin-neuraminidase protein permits the release of the virion. Image courtesy of Ainhoa Calero.

The viral cycle of SeV can be divided into five consecutive stages as represented in Figure 2. (1) Attachment and entry: The HN protein binds to sialic acid residues on the plasma membrane, facilitating viral attachment [10,25]. The F protein induces fusion between the viral envelope and the host membrane, allowing the viral nucleocapsid to enter the cytoplasm [31,32,33,34]. (2) Transcription and translation: Once inside the cytoplasm, the viral genome (ssRNA-) is transcribed into mRNA by the L protein without nuclear integration. Each gene is independently transcribed into a monocistronic mRNA. Host ribosomes efficiently translate these mRNAs into structural (N, P, M, F, HN and L) and non-structural proteins (V, W, C’, C, Y1, Y2, and X) [33,35]. (3) RNA replication: After sufficient viral protein production, the L protein synthesizes a complementary positive-sense RNA (antigenome), which serves as a template for producing new viral genomes (ssRNA-) [36,37,38]. Occasionally, L protein experiences premature termination or polymerase stuttering at specific regions of the genome, producing copyback and internal deletion RNA molecules that lack the full-length sequence of the original viral genome, known as defective-interfering (DI) genomes [39]. (4) Assembly: Newly synthesized viral proteins and genomes assemble in the cytoplasm, particularly at the Golgi apparatus [33]. The N protein encapsulates the viral genome to form nucleocapsids [40]. (5) Budding and release: The assembled nucleocapsids migrate to host cell membrane regions enriched in viral glycoproteins (F and HN) [41], where the M protein coordinates the budding process [42]. The newly formed viral particles are released from the host cell by budding. Neuraminidase activity of the HN protein cleaves sialic acid residues, enabling the release of virions from the host cell and preventing re-attachment to the cell surface [43,44].

## 3. Sendai Virus as a Viral Vector

SeV has been studied as a recombinant viral vector since 1990, following an innovative strategy that allowed the rescue of the rabies virus from cDNA in 1994, called reverse genetics [45,46]. SeV has a simple genome with dispensable genes for gene expression (F, HN, M, P and V) that allows replacement with therapeutic foreign genes [47]. In the first generation of rSeVv, exogenous cDNA was inserted between the 3′ end and the N gene [48]. These rSeVv were replicative and expressed the exogenous component when grown in fertilized chicken eggs [49]. For medical and other practical applications, replication-defective rSeVv with deletion in the F gene (ΔF/SeV) was developed as the second generation [50]. To date, several rSeVv have been examined as viral vector-based vaccines [10,12], cancer therapy [51], gene therapy, and regenerative medicine [47,52,53]. In this review, we focus on SeV as an emerging viral vector-based vaccine platform for animal diseases.

### 3.1. Generation and Rescue of Sendai Virus Vectors

One concern with paramyxovirus-based vectors is the risk of generating infectious viral progeny [54,55]. Thus, second-generation rSeVv have been engineered by deleting V, F, M, HN or P genes. Initially, the V gene was deleted (∆V/SeV) [28], which diminished, but did not eliminate cytopathogenicity and replication, thereby yielding a replication-competent vector and a non-fully attenuated one. Thus, the next step consisted of deleting the structural viral genes to create a non-propagating and non-transmissible versions. Initial efforts were focused on deleting the F gene (ΔF/SeV), replacing it with the enhanced green fluorescent protein (eGFP) gene [50]. Similar strategies were used to generate ∆HN/SeV, ∆M/SeV [56,57], and ∆P/SeV vectors [58]. Double deleted (∆M∆F and ∆F∆HN/SeV) [56,57] and triply deleted (∆M∆F∆HN/SeV) [59] rSeVv were also generated [13]. Moreover, rSeVv with mutations introduced into the F and M genes was also generated [60].

For rSeVv rescue, reverse genetics involves co-transfecting host cells with DNA-plasmids encoding the SeV antigenome, N, P, and L genes, deleted rSeVv gene plasmids and T7 polymerase [46,61]. Since no T7 polymerase is present in eukaryote cells, it was commonly supplied by T7-expressing Vaccinia virus, driving high transcription levels. Cell lines stably expressing T7 polymerase were developed as alternatives [62,63]. Newer SeV systems use optimized T7 promoters without incorporation of non-templated G residues, eliminating the need for Vaccinia virus or T7-expressing cell lines [64]. After rescue, second-generation rSeVv propagation requires helper systems, such as co-supply or cell lines/embryonated eggs expressing the deleted SeV proteins. Using these helper systems, second-generation rSeVv can be propagated in both chicken eggs and mammalian cell lines, yielding high viral titers [65,66]. SeV with mutations introduced into the F and M genes [60] can be propagated in human embryonic kidney 293 cells with SV40 large T antigen (HEK293T) without helper systems [8,64,67]. Once propagated, these virions are infectious and capable of expressing viral or foreign genes, but progeny virions are non-infectious, preventing viral spread.

### 3.2. Biodistribution of Sendai Virus Vectors

rSeVv can infect in vitro and in vivo multiple mammalian cell types, with a primary replication site in the respiratory epithelium [8,68,69]. The F protein requires cleavage into two subunits (F1 and F2) to become biologically active, a process initiated by host-specific proteases such as tryptase Clara, predominantly found in the respiratory tract [68,69]. Other cell types are also permissive to SeV infection [68,70], including retinal epithelium [71,72], hepatocytes [73], hematopoietic stem cells [74,75], monocytes, macrophages, dendritic cells [76], fibroblasts, endothelial, muscle and neuronal cells [50,52,67]. The widespread susceptibility of these cells is partly due to the universal presence of sialylated receptors in animal cells, along with evidence suggesting the existence of a ubiquitous secondary receptor crucial for SeV-mediated membrane fusion [33,77].

Common inoculation routes of rSeVv include intranasal [5,66], intratracheal [78,79,80], intramuscular [6], subcutaneous, intraocular and sublingual [81], with intranasal being the most immunogenic route [80,82].

### 3.3. Foreign Gene Expression of Sendai Virus Vectors

rSeVv can accommodate foreign gene/s of large size (~5 kB) [61,82], with robust, transient, and adjustable transgene expression [8,33,83,84]. The positioning of the foreign gene directly affects vector titers during propagation, with higher titers achieved when the gene is inserted closer to the 5′ trailer sequence [85]. Moreover, gene position relative to the 3′ end influences transgene expression and the induction of antigen-specific immune responses [85,86].

The first inoculation typically yields the highest transgene expression, while subsequent doses are reduced by pre-existing adaptive immune responses [18,47]. However, SeV high infection efficiency still allows sufficient therapeutic levels even with reduced expression after repeated doses [47,83].

### 3.4. Stimulation of Innate Immune Response by Sendai Virus Vectors

SeV is a strong activator of the innate immunity [39] and plays a key role in early protection against viral diseases [8,67]. SeV infection triggers the upregulation of Toll-like receptors (TLR), such as TLR2/6, TLR3, and TLR7, activating the interferon (IFN) pathway and promoting the release of pro-inflammatory cytokines such as IL-2 and IL-6 [24,67,87,88,89,90,91], involved in generating Th1 and Th2-type adaptive responses. SeV, particularly the long copyback dsRNA DI genomes generated during SeV replication, activate the retinoic acid-inducible gene I (RIG-I) pathway, which is responsible for inducing the IFN-β-mediated response [8,39,92,93]. Activation of the RIG-I pathway can also induce the upregulation of IFN-induced protein with tetratricopeptide repeats 2 protein (IFIT2), a pathogen sensor and effector molecule against viral infections [94,95]. Additionally, SeV infection stimulates TRIM family proteins, which are also involved in the innate immune response [96]. In primary pediatric bronchial epithelial cells (WD-PBEC), SeV infection induced high levels of pro-inflammatory cytokines/chemokines, such as IL-6 and Regulated upon Activation, Normal T-Cell Expressed and Presumably Secreted (RANTES), which is particularly interesting from a vaccine perspective. WD-PBEC infected with SeV also showed high levels of the anti-inflammatory cytokine IL-10, an anti-inflammatory cytokine. However, in vivo studies have not demonstrated that IL-10 interferes with the activation of the innate or adaptive immune response. Additionally, SeV infection activates Janus kinase/signal transducers and activators of transcription (JAK-STAT) signaling through interferon-α/β receptor (IFNAR) in U937 cells [97], responsible for producing a wide range of pro-inflammatory cytokines [98]. In ovine skin fibroblasts (OSF), SeV stimulated the upregulation of IFN-stimulated genes (ISGs), such as ovine BST2 (OBST2/Tetherin), tripartite motif-containing protein 5 alpha (TRIM5α), catalytic polypeptide-like 3 (APOBEC3/A3Z1) and SAM domain and HD domain-containing protein 1 (SAMHD1), generating a robust antiviral state [8].

SeV can also induce cell apoptosis by activating the IFN regulatory factor 3 (IRF-3) signaling pathway. IRF-3 mediates the activation of Bax in the HT1080-derived cell line, as well as enhancing p53 signaling in airway epithelial cells [99]. The induction of cell apoptosis can inhibit viral persistent infections [100] and apoptotic cells are efficiently exploited by antigen-presenting cells (APCs) to activate T-cell responses [101]. This complex innate immune response that occurs in SeV-infected cells generates a strong antiviral state that reduces the replication of multiple viruses, making SeV an optimal choice as a viral vector-based vaccine against animal viruses.

### 3.5. Stimulation of Adaptive Immune Response by Sendai Virus Vectors

SeV infection promotes the secretion of cytokines such as IL-2, IL-6, and TNF-α, activating specific Th1 and Th2 immune responses, both necessary for the control of viral replication [93,102,103,104]. Intranasal inoculation induces robust antigen-specific neutralizing serum IgG and mucosal IgA [5,105,106] with a boosting effect [6,7,80], as well as strong T helper [107,108,109] and cytotoxic T CD8+ cells (CTLs) responses [108,110,111]. However, preexisting antibodies against SeV in animals in contact with infected mice could be an obstacle to rSeVv-based T-cell responses induction [112]. Indeed, seropositive individuals to respiratory syncytial virus (RSV) were capable of neutralizing the rSeVv and hindering the RSV transgene expression [10,113]. Interestingly, intranasal SeV administration has conferred a high level of antigen-specific CTL response in the presence of preexisting systemic anti-SeV antibodies [81,112]. Other Paramyxoviruses, such as HPIV-1 and BPIV3, can have similar protein sequences, resulting in cross-reactivity with SeV for both cellular and humoral activities [114,115]. Therefore, it is important to determine whether pre-existing antibodies directed against SeV or related viruses will affect immune responses elicited by rSeVv in animal species.

## 4. Sendai Virus as a Vaccine Platform Against Animal Diseases

Several features of rSeV, including the absence of viral genome integration [33], high levels of foreign gene expression [8,18], robust activation of innate and cellular immune responses [67], and low production costs [65,66], make rSeVv ideal candidates for developing vaccination strategies in animal species. Currently, rSeVv are being tested against influenza A virus, FMDV, SIV and SRLV (Table 1), with promising results.

### 4.1. Sendai Virus Vector as a Vaccine Against Influenza

GP42- rSeVv vector encoding the hemagglutinin (HA) gene from influenza A/Puerto Rico/8/1934 (PR/8) (GP42-SeV-H1) was generated as the first influenza rSeVv-based vaccine prototype [5] (Table 1). GP42-SeV-H1 exhibited a high transgene expression on the cell surface of African Green Monkey Kidney Fibroblast Cells (CV-1 cells) in vitro [5,119]. C57BL/6 mice were intranasally immunized with GP42-SeV-H1, leading to the production of HA-specific IgG and IgA antibodies in sera and mucosal sites without visible signs of disease. Sera from immunized mice exhibited homologous hemagglutination inhibition (HAI) against influenza A virus. Additionally, immunized animals were completely protected against intranasal challenge with a lethal dose of homologous influenza A virus [5,119]. While GP42-SeV-H1 demonstrated strong homologous protection against influenza A, the ultimate goal for many years has been to develop a universal influenza vaccine capable of providing protection against different serotypes.

An influenza vaccine based on a rSeVv (ΔF/SeV) encoding the M2 gene derived from H5N1 avian influenza virus (SeV/ΔF/H5N1M2) was generated [81] (Table 1) and administered in pigs twice via intramuscular or intranasal routes. Intramuscular inoculation of SeV/ΔF/H5N1M2 induced an antibody response to the extracellular domain of the M2 protein (M2e), with only a moderate boosting effect. Interestingly, the intranasal route induced moderate specific antibody titers that cross-reacted with M2e derived from different avian, swine, and human influenza viruses. To demonstrate heterologous protection against different subtypes of influenza, C57BL/6 mice were inoculated with SeV/ΔF/H5N1M2 twice via intramuscular, intranasal, subcutaneous, intraocular, or sublingual routes and then intranasally challenged with H3N2 influenza virus. SeV/ΔF/H5N1M2 did not provide cross-protection against the heterologous influenza virus. Therefore, antibodies to M2e are not immunologically efficient against heterologous influenza viruses [81]. Further studies are needed to identify the foreign genes inserted into the rSeVv that can confer universal protection against different influenza serotypes.

### 4.2. Sendai Virus Vector as a Vaccine Against Foot and Mouth Disease

rSeVv-based vaccines against FMDV have been tested in guinea-pigs inoculated intracardially with allantoic fluid containing SeV (WIC 12,827 strain) and subcutaneously with inactivated FMD vaccine [120]. SeV induced IFN responses immediately, peaking at 5 h, and immunized animals were protected against intradermal challenge with three different FMDV strains (O 1 BFS 1860, A 5 Eystrup and C Neville). However, no antibody production was demonstrated when SeV was administered at the same time as the inactivated FMD vaccine [120]. Therefore, this study supported the generation of a recombinant ΔF/SeV vector encoding the capsid precursor polypeptide (P1) of FMDV serotype O (rSeV-P1) [6] (Table 1), as a prototype FMD vaccine. BALB/c mice were immunized intramuscularly twice with different doses of rSeV-P1 (2^8^ and 2^9^ hemagglutinin antigens (HA)/mouse) or a commercial inactivated FMD vaccine. Four weeks post-booster, mice were challenged with an intraperitoneal injection of virulent serotype O FMDV (O/ES/2001 strain). rSeV-P1 induced high levels of anti-FMDV antibodies with neutralizing activity. Antibody levels significantly increased after the rSeV-P1 booster in a dose-dependent manner and inhibited the replication of FMDV in the sera after FMDV challenge. High doses of rSeV-P1 conferred partial homologous protection against challenge, but it was still lower than the commercial FMD vaccine, showing a lower level of FMDV RNA in the spleen [6]. Therefore, further studies are needed to develop a Sendai virus-based vaccine that provides greater protection against FMDV compared to the inactivated FMD vaccine.

### 4.3. Sendai Virus Vector as a Vaccine Against Animal Retroviruses

#### 4.3.1. Sendai Virus Vector as a Vaccine Against Simian Immunodeficiency Virus

The protective efficacy of rSeVv expressing SIV antigens or CTL epitope-coding peptides has been extensively evaluated in macaque models of SIV infection [7,108,109,111,116,117,118,121,122,123,124,125]. While most of these studies aimed to explore vaccines against the human immunodeficiency virus (HIV) due to its similarities with SIV [126], a closer look at rSeVv, developed as vaccines specifically targeting SIV, is worth further exploration. These studies established diverse immunization protocols, typically involving an initial intramuscular DNA priming step followed by one or more intranasal booster doses with either replication-competent (ΔV/SeV) [28] or replication-defective (ΔF/SeV) [50] rSeVv encoding different SIV genes.

First, ΔV/SeV encoding SIV-*Gag* gene (SeV/SIV-*Gag*) was generated [7] (Table 1). Macaques were immunized intranasally thrice with SeV/SIV-*Gag*, exhibiting high titers of anti-SeV antibodies, with rapid increases in their levels in the second and third immunizations, suggesting a boosting effect. However, no anti-SIV-*Gag* antibodies and *Gag*-specific T-cells were observed. Interestingly, after intravenous challenge with SIVmac239 [127], no significant decrease in peripheral CD4+ or CD8+ T-cell numbers and a marked reduction in plasma viral load were detected [7]. Although these results indicated early protection against SIV, the immunological correlations for protection remained unclear. Therefore, this study supported the development of new immunization protocols against SIV based on rSeVv-based vectors.

New heterologous immunization regimens were developed, incorporating intramuscular priming with plasmids encoding SIV or simian-human immunodeficiency virus (SHIV) genes, followed by an intranasal booster with recombinant ΔV/SeV or ΔF/SeV vectors encoding the SIV-*Gag* gene [108,121] (Table 1). Macaques exhibited a robust *Gag*-specific CD8+ T-cell response after booster and controlled viremia without acute CD4+ T-cell depletion after SHIV89.6PD [128] or SIVmac239 [127] challenge, suggesting partial protection against SIV [108,111,117,121,123]. However, the immunization regimen with the ΔF/SeV vector failed to contain the infection with different SIV strains, carrying multiple *Gag* CTL escape mutations [123]. Additionally, systemic *Gag*-specific CD8+ T-cell responses were maintained longer in the ΔV/SeV-*Gag*-boosted macaques, suggesting that replication-competent rSeVv are more immunogenic over the long term [124].

To determine the long-term protection efficiency of these heterologous immunization regimens, five macaques showing vaccine-based control of SIVmac239 replication [111] were studied for the following years [122,123]. While three of these animals controlled the SIV infection without additional mutations in the SIV provirus for more than 3 years [123], the other two animals showed SIV mutations, leading to viral evasion from three epitope-specific CTL responses. Accumulation of these multiple escape mutations resulted in the reappearance of plasma viremia around week 60 after challenge [122]. Therefore, the sequential accumulation of multiple CTL escape mutations can lead to SIV evasion of immune control, with significant implications for vaccine design. This underscores the importance of eliciting long-term and broad, multi-epitope CTL responses to effectively suppress SIV replication.

Subsequently, other SIV genes were evaluated for the generation of new rSeVv-based vaccines. ΔV/SeV encoding HIV-1_NL4-3_ *Tat* [116] and ΔF/SeV encoding SIV *Gag* or *Vif*/*Nef* genes [118] were generated (Table 1). *Gag* and *Vif*-specific CD8+ T-cell responses controlled SIV replication, whereas *Tat* and *Nef* T-cell responses failed to control SIV replication [116,118]. Therefore, *Gag* and *Vif* genes could be the most promising candidates for insertion in rSeVv-based vaccines.

rSeVv have also been studied as therapeutic vaccines against SIV. Previously immunized and challenged macaques [116,121] were again intranasally immunized with ΔV/SeV-*Gag* or ΔF/SeV-*Gag* [109]. rSeVv distribution after therapeutic immunization was localized in the nasal mucosa and regional primary lymph nodes. Immunized animals showed rapid expansion of SeV-specific T-cell responses and *Gag*-specific CD4+ and CD8+ T-cell responses, suggesting a therapeutic effect in the chronic phase of the disease [109]. To compare the therapeutic effect between antiretroviral therapy (ART) and rSeVv, twelve macaques received ART after intravenous challenge with SIVmac239 [127] and six of them were immunized intranasally with ΔF/SeV-*Gag* and ΔF/SeV-*Vif* [125]. Immunized animals were able to enhance predominantly *Gag*/*Vif*-specific CD8+ T-cell responses and controlled SIV viremia during ART, but showed viremia rebound after ART cessation. However, replication of autologous PBMC-derived SIV was neutralized by the anti-SIV efficacy of CD8+ cells induced by ΔF/SeV-*Gag*/*Vif* immunization under ART [125]. Therefore, these studies suggest the potential combined use of rSeVv-based vectors as therapeutic vaccines for animal retroviruses.

#### 4.3.2. Sendai Virus Vector as a Vaccine Against Small Ruminant Lentiviruses

The innate immune response induced by the ΔF-M/SeV vector encoding the green fluorescent protein gene (SeV-GFP) was characterized in ovine cells in vitro. Ovine alveolar macrophages (AM), blood-derived macrophages (BDM), and OSF demonstrated permissiveness to in vitro infection with SeV-GFP. Infected AM, BDM, and OSF exhibited partial restriction against SRLV, serotype A (strain EV1) [129] infection. Interestingly, SeV-GFP infection also triggered the secretion of antiviral factors in AM with paracrine effects. Additionally, SeV-GFP-infected myeloid cells (AM and BDM) showed a macrophage M1-like differentiation, along with APOBEC/A3Z1 upregulation, suggesting the induction of antiviral responses. SeV-GFP also induced robust innate immune stimulation in OSF, characterized by upregulation of RIG-I and OBST2 [67].

Therefore, these results supported the generation of a recombinant SeV-GFP encoding SRLV *gag*-P25 gene (rSeV-GFP-P25), derived from SRLV genotype A (strain EV1), as a vaccine prototype against SRLV (Table 1) [8]. rSeV-GFP-P25 showed efficient and transient transgene expression in SeV-infected OSF in vitro and in the ciliated epithelial cells and submucosal macrophages/dendritic cells of the nasal cavity of a lamb infected intranasally. Moreover, rSeV-GFP-P25 induced a robust stimulation of the innate immune response in infected OSF, triggering the upregulation of IFN-β and several ISGs, including OBST2, APOBEC/A3Z1 and, to a lesser extent, SAMHD1. rSeV-GFP-P25 promoted a high homologous restriction to SRLV strain EV1 [129] infection in OSF [8]. Therefore, these results justify further investigation into the use of rSeVv-based vaccines for immunization against SRLV.

## 5. Conclusions and Future Directions

rSeVv represents a versatile and promising platform for vaccine development. Their capacity to elicit strong immune responses, combined with a favorable safety profile, positions them as valuable candidates in the prevention and control of infectious diseases. Building on these encouraging findings, rSeVv-based vaccines warrant further exploration for the development of broad-spectrum vaccines targeting animal viruses. The strong innate immune activation triggered by rSeVv is particularly important for early viral control and supports their potential application in therapeutic settings, where they may contribute to reducing lesion severity and viral load. In addition, the induction of long-lasting, antigen-specific CD8^+^ T-cell responses underscores their value as prophylactic vaccines, especially in addressing currently unmet needs in the control of animal retroviruses. Although rSeVv-based formulations have demonstrated strong homologous protection against viruses such as influenza A, FMDV, SIV, and SRLV, achieving heterologous protection remains a key challenge, likely influenced by the nature of the antigenic insert. Importantly, rSeVv can accommodate multiple gene inserts, offering a platform for the development of multivalent vaccines targeting diverse viral genotypes or serotypes. Future research should aim to enhance cross-protective efficacy, overcome anti-vector or pre-existing immunity, assess therapeutic efficacy through antiviral activity, and ensure scalability for deployment in practical field applications. The development of rSeVv-based vaccines could revolutionize veterinary health by providing effective and long-lasting protection against infectious diseases.

## Figures and Tables

**Table 1:** Research rSeVv-based vaccines against influenza A, foot and mouth disease virus (FMDV), simian immunodeficiency virus (SIV) and small ruminant lentiviruses (SRLV).

Pathogen	Vaccine Name	SeV Vector	Inserted Gene	Insertion Site	Host	References
Influenza A	GP42-SeV-H1	GP42-SeV	HA (A/PR/8 (H1N1))	M-F	C57BL/6 mice	[5]
	SeV/ΔF/H5N1M2	ΔF/SeV	M2	Not specified	Guinea pigs and C57BL/6 mice	[81]
FMDV	rSeV-P1	ΔF/SeV	FMDV-P1	N-P	BALB/c mice	[6]
SIV	SeV/SIV-Gag	ΔV/SeV	SIV-*Gag*	5’-N	Cynomolgus and rhesus macaques	[7]
	SeV-Tat	ΔV/SeV	HIV-*Tat*	5’-N	Rhesus macaques	[116]
	F(-)SeV-Gag	ΔF/SeV	SIV-*Gag*	Not specified	Rhesus macaques	[108]
	F(-)SeV-Gag236-250-EGFP	ΔF/SeV	SIV-*Gag*236-250-EGFP	Not specified	Burmese rhesus macaques	[117]
	F(-)SeV-Vif	ΔF/SeV	*Vif*-opt	Not specified	Burmese rhesus macaques	[118]
	F(-)SeV-Nef	ΔF/SeV	*Nef*-G2A	Not specified	Burmese rhesus macaques	[118]
SRLV	rSeV-GFP-P25	ΔF-M/SeV	*Gag*-P25	N-P	Lambs	[8]

## Abbreviations

The following abbreviations are used in this manuscript:

AM	Alveolar macrophages
APCs	Antigen-presenting cells
APOBEC3/A3Z1	Catalytic polypeptide-like 3
ART	Antiretroviral therapy
BDM	Blood-derived macrophages
BPIV3	Bovine parainfluenza virus 3
CTLs	Cytotoxic T CD8+ cells
CV-1 cells	African Green Monkey Kidney Fibroblast Cells
F	Fusion
FMD	Foot-and-mouth disease
FMDV	Foot-and-mouth disease virus
HA	Hemagglutinin antigens
HAI	Hemagglutination inhibition
HIV	Human immunodeficiency virus
HN	Hemagglutinin-neuraminidase
HPIV1	Human parainfluenza virus 1
HPIV3	Human parainfluenza virus 3
IFIT2	Interferon-induced protein with tetratricopeptide repeats 2 protein
IFN	Interferon
IFNAR	Interferon-α/β receptor
IRF-3	Interferon regulatory factor 3
ISGs	Interferon-stimulated genes
JAK-STAT	Janus kinase/signal transducers and activators of transcription
L	Large polymerase
M	Matrix
M2e	M2 protein
N	Nucleocapsid
OBST2/Tetherin	Ovine BST2
OSF	Ovine skin fibroblasts
P	Phosphoprotein
RANTES	Regulated upon activation, normal T-cell expressed and presumably secreted
RIG-I	Retinoic acid-inducible gene I
rSeVv	Recombinant Sendai virus vectors
RSV	Respiratory syncytial virus
SAMHD1	SAM domain and HD domain-containing protein 1
SeV	Sendai virus
SHIV	Simian-human immunodeficiency virus
SIV	Simian immunodeficiency virus
SRLV	Small ruminant lentiviruses
TLR	Toll-like receptors
TRIM5α	Tripartite motif-containing protein 5 alpha
WD-PBEC	Primary pediatric bronchial epithelial cells
